# Mitochondrial ROS and base excision repair steps leading to DNA nick formation drive ultraviolet induced-NETosis

**DOI:** 10.3389/fimmu.2023.1198716

**Published:** 2023-06-07

**Authors:** Dhia Azzouz, Nades Palaniyar

**Affiliations:** ^1^ Translational Medicine, Peter Gilgan Center for Research and Learning, The Hospital for Sick Children, Toronto, ON, Canada; ^2^ Department of Laboratory Medicine and Pathobiology, University of Toronto, Toronto, ON, Canada; ^3^ Institute of Medical Sciences, Faculty of Medicine, University of Toronto, Toronto, ON, Canada

**Keywords:** neutrophil extracellular trap formation, UV-iradiation, mitochondrial ROS (mitoROS), oxidation of DNA, base excision repair (BER), DNA nick formation, chromatin decondensation

## Abstract

Reactive oxygen species (ROS) is essential for neutrophil extracellular trap formation (NETosis), and generated either by NADPH oxidases (e.g., during infections) or mitochondria (e.g., sterile injury) in neutrophils. We recently showed that ultraviolet (UV) radiation, a sterile injury-inducing agent, dose-dependently induced mitochondrial ROS generation, and increasing levels of ROS shifted the neutrophil death from apoptosis to NETosis. Nevertheless, how ROS executes UV-induced NETosis is unknown. In this study, we first confirmed that UV doses used in our experiments generated mitochondrial ROS, and the inhibition of mitochondrial ROS suppressed NETosis (Mitosox, SYTOX, immunocytochemistry, imaging). Next, we showed that UV irradiation extensively oxidized DNA, by confocal imaging of 8-oxyguanine (8-oxoG) in NETs. Immunofluorescence microscopy further showed that a DNA repair protein, proliferating cell nuclear antigen, was widely distributed throughout the DNA, indicating that the DNA repair machinery was active throughout the genome during UV-induced NETosis. Inhibition of specific steps of base excision repair (BER) pathway showed that steps leading up to DNA nick formation, but not the later steps, suppressed UV-induced NETosis. In summary, this study shows that (i) high levels of mitochondrial ROS produced following UV irradiation induces extensive oxidative DNA damage, and (ii) early steps of the BER pathway leading to DNA nicking results in chromatin decondensation and NETosis. Collectively, these findings reveal how ROS induces NOX-independent NETosis, and also a novel biological mechanism for UV irradiation- and -mitochondrial ROS-mediated NETosis.

## Introduction

Neutrophil extracellular trap formation (NETosis) is a unique form of cell death involving multiple steps that results in chromatin decondensation and NET release (1–5). Whether NETosis and apoptosis can occur simultaneously in neutrophils has long remained a mystery. However, we uncovered that UV could induce concomitant NETosis and apoptosis in the same neutrophil (6). While UV-induced apoptosis is well understood after decades of extensive studies, our knowledge about the inner workings of the newly discovered UV-induced NETosis is still limited. We have recently uncovered that mitochondrial ROS (mitoROS) is necessary for UV-induced NETosis (7). However, how this ROS participates in UV-induced NETosis pathway is unknown.

ROS generation is a cardinal step in NETosis; its importance has been known since the discovery of NETosis, several decades ago (1, 2). Nevertheless, how ROS regulates NETosis is not clearly established. Neutrophils generate ROS either by NADPH oxidase (NOX) or *via* mitochondria. In general, microbial infections activate NOX-dependent pathway (e.g., LPS, bacteria) (2, 8), whereas sterile injury often generates mitoROS and activates NOX-independent NETosis (e.g., uric acid crystals, autoimmune complexes, and UV radiation) (6, 7, 9, 10). During both types of NETosis, chromatin is decondensed and covered with cytotoxic peptides, and enzymes such as myeloperoxidase (MPO) and other neutrophil proteases (11, 12). We have recently uncovered that ROS generated by NOX oxidizes neutrophil DNA and the repair pathway is essential for driving spontaneous or agonist-induced NOX-dependent NETosis (13, 14). By contrast, how mitoROS regulates UV-mediated NOX-independent NETosis is unknown. Oxidative DNA damage is repaired *via* base excision repair (BER) pathway (15, 16). In this study, we aim to determine whether mitoROS exerts its effect in UV-induced NETosis through the activation of BER.

## Methods

### Neutrophil isolation from human peripheral blood

This study was approved by the Research Ethics Board of the Hospital for Sick Children. Blood was drawn from healthy donors and deposited into K2 EDTA blood collection tubes. PolymorphPrep was used to isolate the neutrophils from blood samples within five minutes of collection. Manufacturer’s instructions were followed with the following key modifications to the protocol. A red blood cell lysis step was carried out using a 0.2% (w/v) NaCl hypotonic solution. The solution was then turned isotonic and buffered by adding an equal volume of 1.6% (w/v) NaCl solution with Hepes buffer (20 nM, pH 7.2). The isolated neutrophils were then resuspended in RPMI medium (Invitrogen) supplemented with Hepes buffer (10 mM, pH 7.2).

### Inducing NETosis and apoptosis using UV

Cells were treated with UV irradiation using a Stratalinker 2400 (Stratagene) machine. Cells were irradiated with 1.92 J/cm^2^ of UVC light to induce NETosis (7). The UV dose used was previously shown to induce NETosis in neutrophils (17).

### SYTOX Green plate reader assay for DNA release analysis

SYTOX Green dye (5 μM; ThermoFisher Scientific) was added to cells suspended in RPMI media + 10 mM Hepes (5 x 10^5^ cells per ml). Cells were then plated on a 96-well plate (100 μl per well). Cells were incubated for 1 hour with inhibitors at 37°C. Media (negative control), UV was used to activate cells. The inhibitors used were APE inh 1 (CRT0044876, Sigma), APE inh 2 (APE1 Inhibitor III, EMD Millipore), PARP1 inh 1 (BSI201, Sigma), PARP inh 2 (PJ34, EMD Millipore), LIG inh (L189, Tocris), Pol δ inh (Aphidicolin, Sigma), Pol β inh (AM-TS23, Tocris) and Proliferating Cell Nuclear Antigen inhibitor (PCNA inh; T2AA, Tocris). The inhibitors were dissolved in DMSO and then diluted in RPMI media to achieve the required concentrations. After being added to the samples, the fluorescence produced by the interaction between SYTOX Green and DNA was measured using a POLARstar OMEGA fluorescence plate reader (BMG Labtech), with excitation at 485 nm and emission at 525 nm, after 240 minutes. The levels of NETosis were determined based on the measured fluorescence, and the NETosis percentage was calculated by dividing the fluorescence reading of each treatment by the reading of cells treated with 1% (v/v) Triton X-100.

### MitoSOX plate reader assay (mitochondrial ROS measurement)

To perform the MitoSOX assay, cells were seeded onto a 96-well plate at a concentration of 1 x 10^6^ cells per ml in a volume of 100 μl. After UV irradiation, 5 µM of MitoSOX was added to each well. The fluorescence resulting from the oxidation of MitoSOX was measured using a POLARstar OMEGA fluorescence plate reader (BMG Labtech), with excitation at 510 nm and emission at 580 nm, after 30 minutes of cell activation.

### DHR123 plate reader assay (NOX-derived ROS measurement)

For the dihydrorhodamine 123 (DHR123) plate reader assay, cells were seeded on a 96-well plate at a concentration of 1 × 10^6^ cells per ml. After UV irradiation, 25 µM of DHR123 was added to each well. Following cell activation, the fluorescence resulting from the oxidation of DHR123 to R123 was measured using a POLARstar OMEGA fluorescence plate reader (BMG Labtech), with excitation at 485 nm and emission at 525 nm, after 240 min.

### Confocal imaging

Cells were plated on a 96-well plate (100 μl, 1 x 10^6^ cell per ml). Cells were incubated for 1 hour with inhibitors at 37°C. Media (negative control) and UV were then used as cell activators. The following inhibitors were utilized in the study: APE inh 1 (Sigma, CRT0044876), APE inh 2 (EMD Millipore, APE1 Inhibitor III), PARP1 inh 1 (Sigma, BSI201), PARP inh 2 (EMD Millipore, PJ34) and LIG inh (Tocris, L189). These inhibitors were dissolved in DMSO and then diluted in RPMI media to reach the desired concentrations before being added to the samples. After the 240-minute mark, the reactions were terminated with 4% (w/v) paraformaldehyde (PFA) and incubated overnight. Subsequently, the cells were permeabilized with 1% Triton X-100 for 25 minutes and blocked with 2.5% (w/v) BSA in PBS for 1 hour. Visualization of DNA was achieved by using DAPI (10 μM; ThermoFisher Scientific) at 1:333 dilution. Mouse anti-PCNA antibody (F-2, Santa Cruz) at a 1:250 dilution was used for probing PCNA, while mouse anti-8-Oxoguanine antibody (MAB3560, Millipore Sigma) at a 1:250 dilution was used for probing 8-oxogGuanine. Olympus IX81 inverted fluorescence microscope equipped with a Hamamatsu C9100-13 back-thinned EM-CCD camera and Yokogawa CSU ×1 spinning disk confocal scan head was utilized for imaging.

### Statistical analyses

GraphPad Prism 7 was used for statistical analysis. One-sample t-test, one-way ANOVA with Dunnett’s post-test, or two-way ANOVA with Dunnett’s post-test was used as appropriate. Groups being compared had similar variance, and error bars indicate ± SEM. A p-value less than 0.05 was considered statistically significant.

## Results

### UV radiation induces mitoROS-dependent NETosis

**Figure 1:**
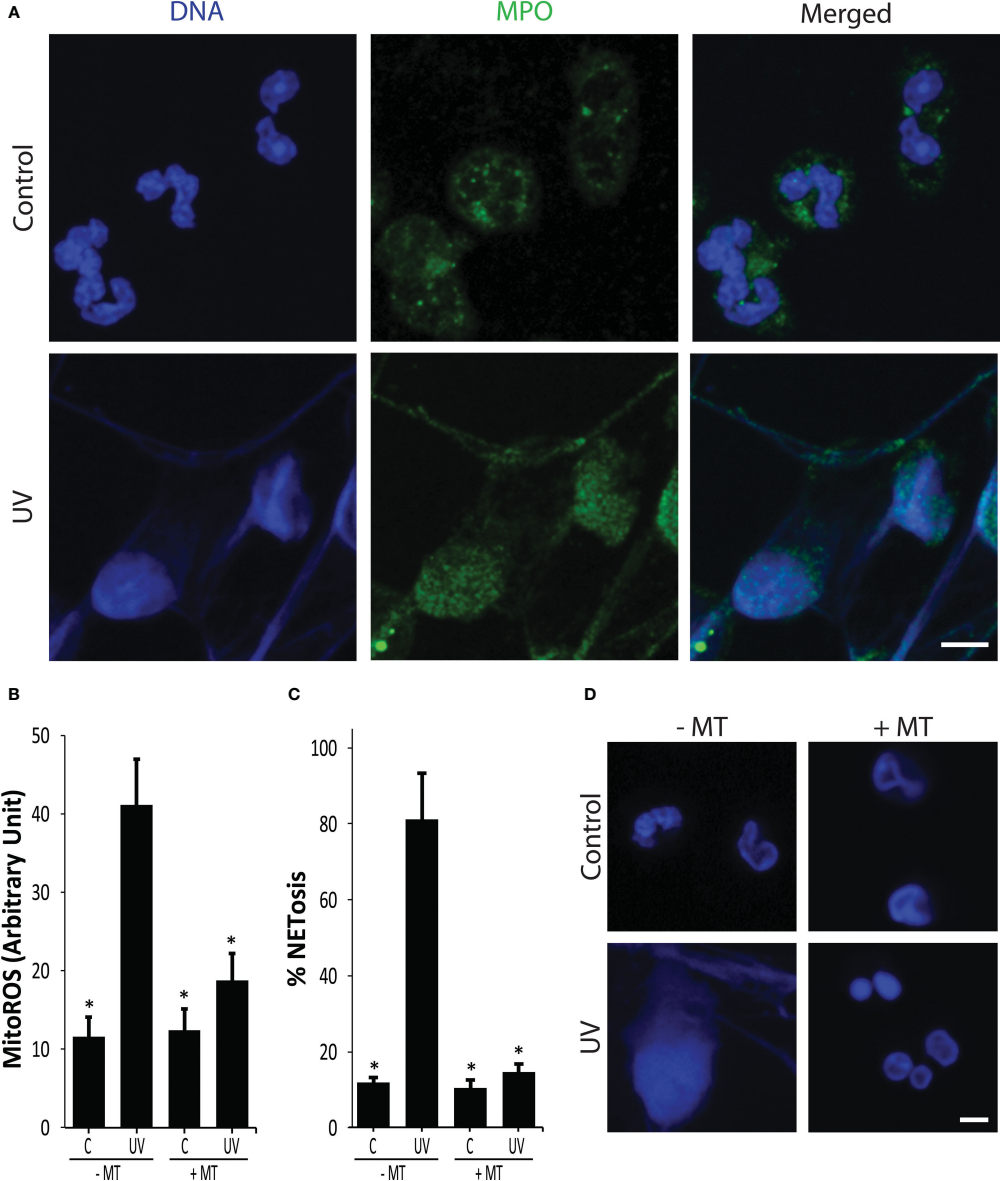
UV induces NETosis in a NOX-independent manner. **(A)** Neutrophils were stained for DNA (blue) and MPO (green). Immunofluorescence confocal imaging shows that MPO colocalizes to DNA, hence 1.92 J/cm^2^ UV induces NETosis. Images are representative of three independent experiments. Scale bar, 10 μm. **(B)** Mito ROS was measured using the MitoSOX plate reader assay. UV (1.92 J/cm^2^) induced mitochondrial-derived ROS (n = 3; error bars represent SEM; *p < 0.05 for comparing with the UV without MitoTempo (MT) sample; Two-way ANOVA with Dunnett’s multiple comparison test). **(C)** DNA release following UV (1.92 J/cm^2^) exposure was measured using SYTOX Green plate reader assay (n = 3; error bars represent SEM; *p < 0.05 for comparing with the UV without MT sample; One-way ANOVA with Dunnett’s multiple comparison test). Cells were preincubated with MitoTempo (100 µM) for 1-h prior to UV treatment. MitoTempo significantly inhibits UV-induced NETosis. **(D)** Neutrophils were incubated with MitoTempo (100 µM) for 1 h and then treated with UV (1.92 J/cm^2^) and incubated for 240 min. Cells were stained for DNA (DAPI, blue). Fluorescence confocal imaging indicates that MitoTempo inhibits UV-induced NETosis. Images are representative of three independent experiments (scale bar, 10 μm).

Our previous study showed that increasing doses of UV irradiation increase mitoROS production and NETosis (7). To confirm that the UV dose used in this study induced NETosis, we first used confocal imaging, and examined chromatin decondensation and MPO colocalization with DNA, a hallmark of NETosis (18, 19). Immuno-confocal imaging showed that NETs were extensively decorated by MPO, indicating that 1.92 J/cm^2^ of UV was inducing NETosis (**Figure 1A**).

In neutrophils, ROS can either be generated by NOX enzyme or by mitochondria, and the source of ROS is a key distinguishing factor between different types of NETosis (2, 20). We used MitoSOX (a mitochondria-specific dye), DHR123 (an intracellular ROS dye), and plate reader assays to determine ROS production and origin. UV exposure (1.92 J/cm^2^) of neutrophils generated mitoROS, but not NOX ROS (**Figures 1B**, **S1**). A mitoROS inhibitor, MitoTempo, significantly reduced mitochondrial ROS generation in neutrophils that further supports UV inducing mitoROS (**Figure 1B**). SYTOX Green (cell impermeable, DNA-binding dye) plate reader assays and confocal imaging results indicate that MitoTempo inhibited UV-induced NETosis (**Figure 1C, D**), and did not induce apoptosis (**Figure S2**). The involvement of mitoROS was further supported using the mitochondrial uncoupler 2,4-dinitrophenol (DNP) (**Figure S3**). The NOX ROS inhibitor diphenyleneiodonium (DPI) was used to determine the involvement of NOX in UV-mediated NETosis. DPI was found to not reduce UV-mediated NETosis, indicating that it is a NOX-independent process (**Figure S4**). Collectively, these results show that UV induces mitoROS-mediated NOX-independent NETosis.

### UV extensively oxidizes neutrophil DNA

**Figure 2:**
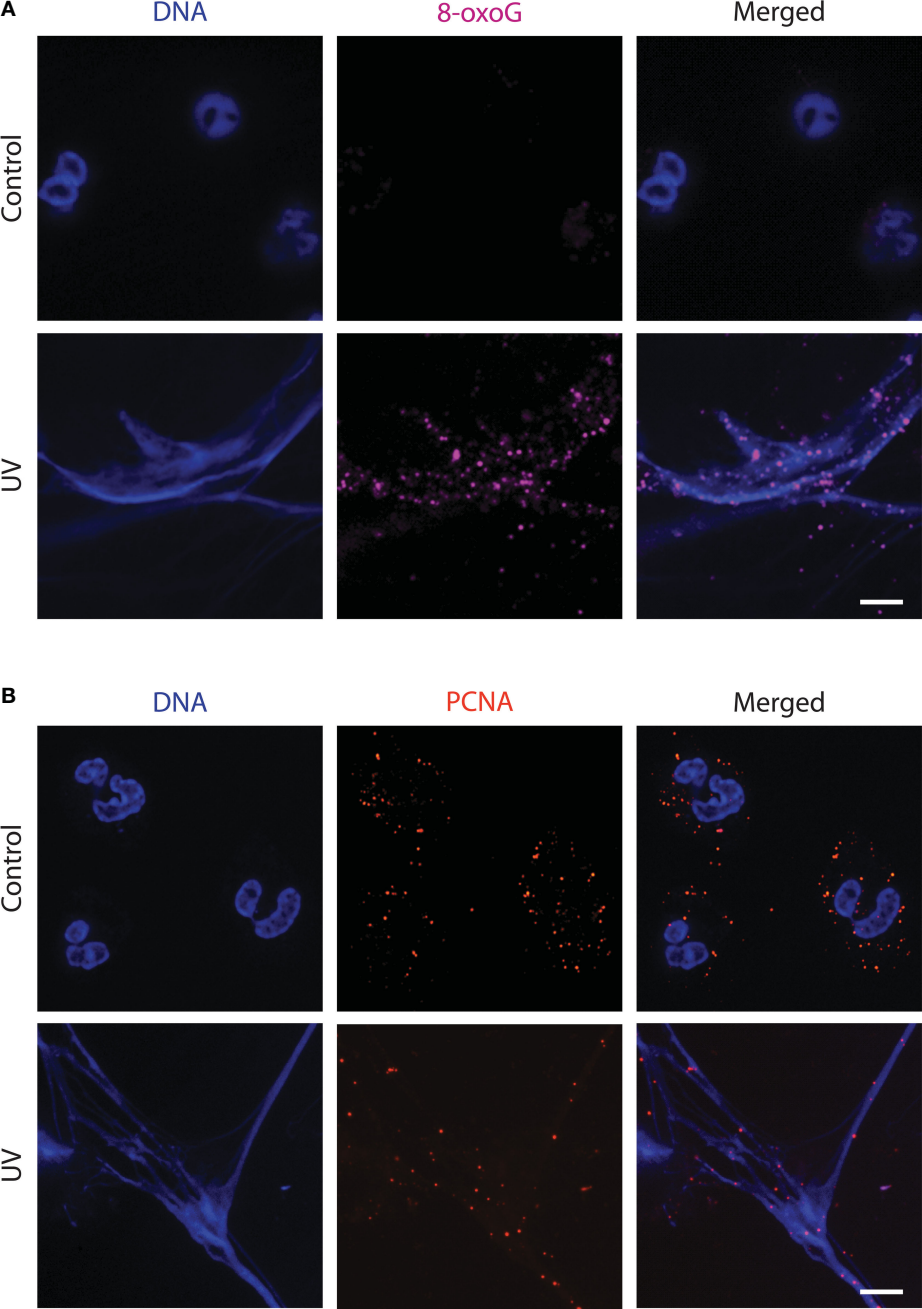
UV-treated neutrophils release NETs that have sustained oxidative DNA damage in the form of 8-oxoG and are decorated with PCNA. **(A)** Neutrophils were treated with media (negative control) or UV (1.92 J/cm^2^) and incubated for 240 minutes. Cells were stained for DNA (blue) and 8-oxoG (magenta). The images are representative of three independent experiments. Scale bar, 10 μm. **(B)** Neutrophils were treated with UV (1.92 J/cm^2^) and incubated for 240 minutes. Cells were stained for DNA (blue) and PCNA (red). The images are representative of three independent experiments. Scale bar, 17 μm.

ROS is the primary endogenous agent responsible for DNA damage, in neutrophils (7, 21). Specifically, ROS oxidizes the ring atoms of purines, such as guanine. The most common form of oxidative DNA damage product is 7,8-dihydro-8-oxoguanine (8-oxoG) (22). To determine the effect of ROS on NET DNA, we irradiated the neutrophils with UV and immunostained them against 8-oxoG. The images showed that UV extensively induced oxidative DNA damage (**Figure 2A**). Hence, the mitoROS generated during UV irradiation induces oxidative damage to neutrophil DNA.

### DNA repair protein Proliferating Cell Nuclear Antigen (PCNA) is present on the NETs

Since significant base oxidation was occurring after UV irradiation, we next determined whether neutrophil engages in DNA repair. The BER pathway serves as the main mechanism for repairing oxidative damage to DNA (15, 23). PCNA is a DNA clamp involved in repair pathways and has been reported to be present in large amounts in the cytoplasm of healthy neutrophils (24). We immunostained for PCNA to detect the presence of this proteins on NET DNA as an indicator of repair machinery assembly during NETosis. Using confocal imaging, we found that PCNA present in the cytoplasm of the resting neutrophils was localized throughout the NET DNA during UV-induced NETosis (**Figure 2B**). Hence, DNA repair is active on NET DNA during UV-induced NETosis.

### Early steps of BER that lead to DNA nick formation are essential for UV-mediated NETosis

**Figure 3:**
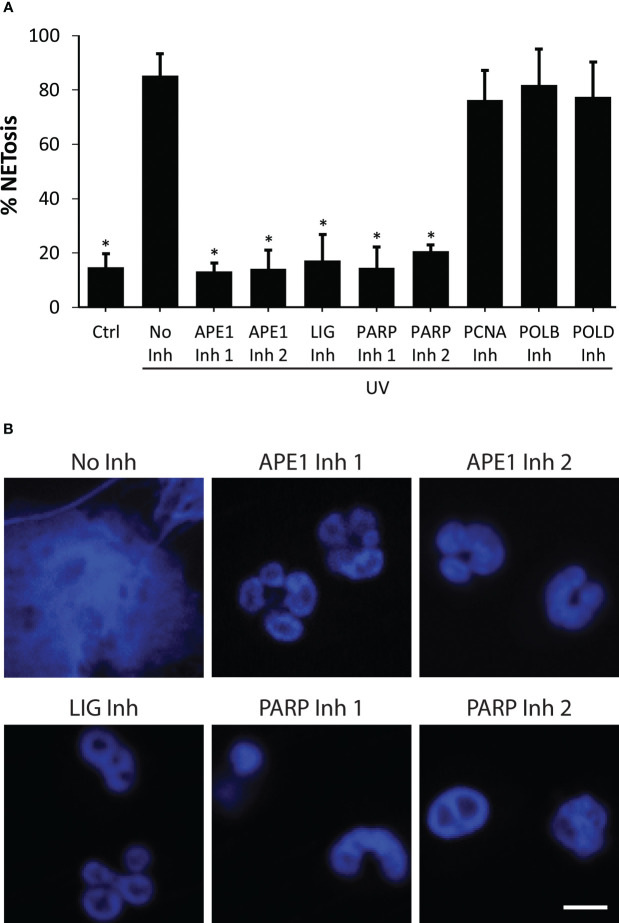
Inhibition of early steps of BER suppresses NETosis induced by UV. **(A)** DNA release from neutrophils following UV irradiation (1.92 J/cm^2^) was measured using the SYTOX Green plate reader assay in the presence or absence of DNA repair inhibitors (Inh). Cells were preincubated with BER inhibitors (APE1 inh 1, CRT0044876 (125 μM); APE1 inh 2, APE1 Inhibitor III (50 μM); PARP1 inh 1, BSI201 (100 μM); PARP inh 2, PJ34 (50 μM), LIG inh, L189 (100 μM), Polymerase β (POLB) inh, AM-TS23 (25 μM); proliferating cell nuclear antigen (PCNA) inh, T2AA (25 μM) or Polymerase δ (POLD) inh, Aphidicolin (50 μM)) for one hour prior to UV treatment. Maximum SYTOX signal obtained 240 minutes after UV irradiation was considered 100% NETosis. The data are presented a mean ± SEM (n = 3; *, p<0.05 compared to the cells treated with UV with no inhibitors; One-way ANOVA with Dunnett’s multiple comparison test). **(B)** Neutrophils were incubated with DNA repair inhibitors for one hour then treated with UV (1.92 J/cm^2^), incubated for 240 minutes, and stained for DNA (blue). The images are representative of three independent experiments. Scale bar, 10 μm.

To determine the importance of DNA repair to UV-induced NETosis, we studied the effects of BER inhibitors using the SYTOX Green assay and confocal imaging. The cells were treated with BER inhibitors, including APE1, PARP1, DNA ligase, PCNA, Polβ, and Polδ, and incubated for one hour. The cells were then treated with media control or UV. Our results uncovered that inhibiting early steps of BER (APE1, PARP1, DNA ligase) stifled UV-induced NETosis, while inhibiting later stages of BER (PCNA, Polβ and Polδ) had no effect (**Figures 3A, B**). These studies reveal that initial DNA strand nicking/chromatin decondensing steps of BER are the steps required for ROS-mediated UV-induced NETosis.

## Discussion

**Figure 4:**
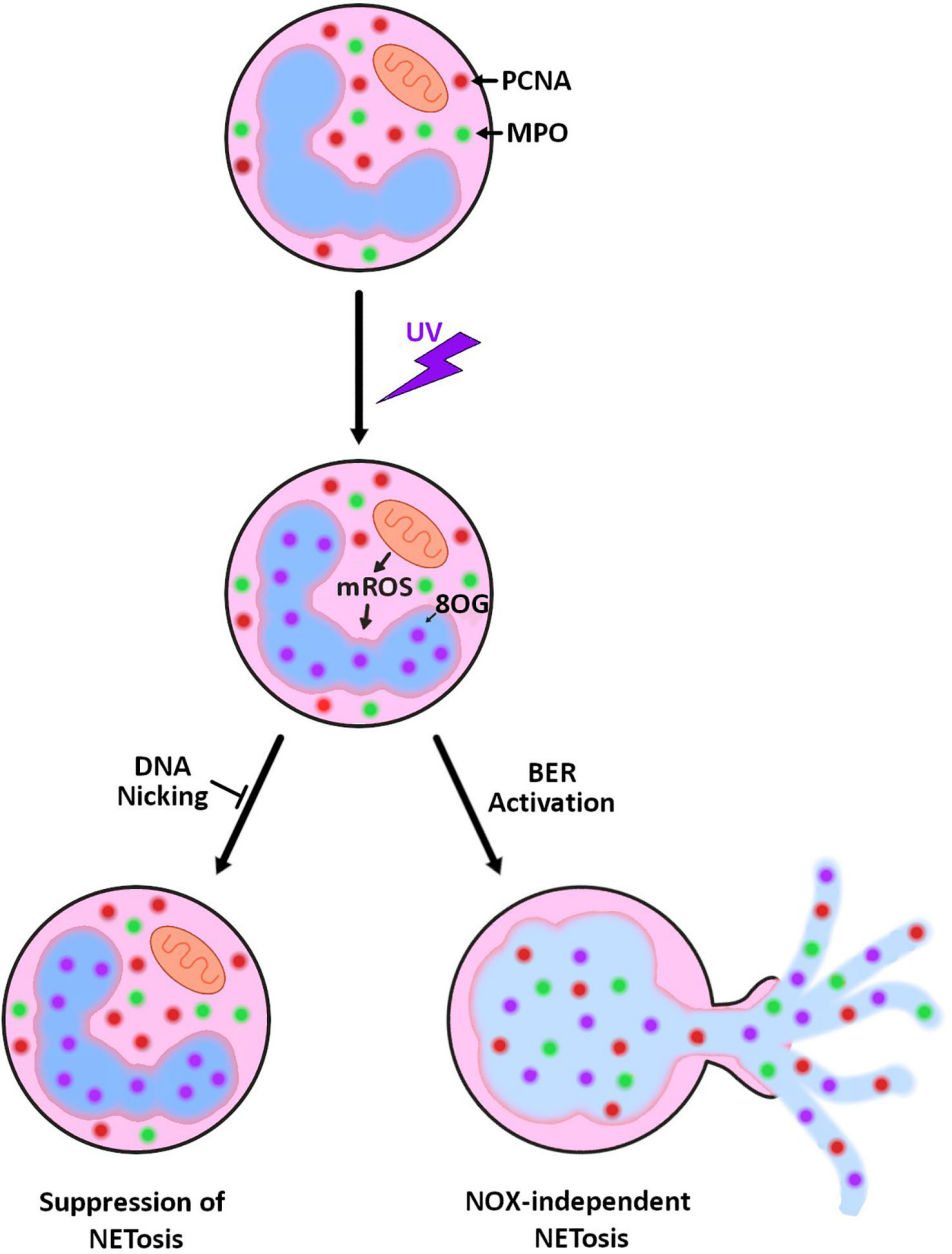
Summary diagram showing the role of mitoROS and DNA repair in UV-induced NETosis. UV irradiation results in increased mitoROS production (mROS), and as a result, increased 8-oxoguanine (8-oxoG) lesions on the DNA. Attempted repair of the damage by base excision repair results in NETosis. This NETosis can be prevented by inhibiting the nicking/unwinding steps of base excision repair.

Neutrophils are terminally differentiated cells with a short lifespan. They contain many pre-made DNA repair proteins but their genome is not actively transcribed or replicated (24–27). Hence, neutrophils carrying the DNA repair machinery is puzzling. We have recently shown that BER activation is necessary for spontaneous and agonist-induced NOX-dependent NETosis activated by PMA, LPS, *Pseudomonas aeruginosa* and *Staphylococcus aureus* (13, 14). Specifically, we uncovered that chromatin unwinding capability of BER is one of the key drivers of NETosis. The current study establishes that DNA strand nicking and chromatin unwinding capabilities of BER are also instrumental in UV-induced NETosis. More specifically, in UV-induced NETosis, mitoROS oxidizes bases, such as guanine to 8-oxoG, which results in the recruitment of BER machinery to the sites of damage. The BER machinery attempts to repair the DNA by unwinding the chromatin, removing the damaged bases and nicking the chromatin. Hence, these DNA nicking and chromatin unwinding are key drivers of UV-induced NETosis (**Figure 4**).

Neutrophils generate ROS either *via* NOX or mitochondria (2, 18). These ROS could cause oxidative damage to its own DNA (13). It is well established that the oxidized bases are removed by DNA glycosylases and the DNA strands are cleaved at the deglycosylated base by APE1 (24–27). This is followed by PARP binding to the single stranded DNA ends and generating poly ADP at the damage sites. These steps lead to the recruitment of the remainder of the repair machinery, which includes DNA ligases (28). The recruitment of the initial 3 enzymes (APE1, PARP, DNA Ligase) at the site of DNA damage is sufficient to cause significant chromatin decondensation and nicking (29). Afterward, PCNA assembles into a trimeric ring around the DNA, facilitating the recruitment of repair DNA polymerases β and δ and other repair protein complexes to complete the DNA repair process (15, 28). Hence, early steps of BER pathway leading to nick formation are critical steps for preventing chromatin unwinding.

In this study, we first confirmed that UV-induced NETosis results in mitochondrial ROS production. We studied cellular ROS using DHR123 dye and mitochondrial ROS using mitoSOX. Our DHR123 experiments showed that PMA, but not UV, induced cellular ROS (**Figure S1**). This was further supported by our DPI (NOX inhibitor) data, which found that PMA-, but not UV-induced NETosis was inhibited by DPI (**Figure S4**). On the other hand, UV induced mitochondrial ROS (as determined by MitoSOX; **Figure 1B**), and UV-induced NETosis was suppressed by MitoTempo (**Figure 1C**) and the mitochondrial uncoupler DNP (**Figure S3**). These data sets indicate that UV induces ROS from the mitochondria and not from NOX. It is possible that UV is generating other forms of ROS as reported by others (30). Neubert et al. and Arzumanyan et al. showed that red, blue and long-wave UV light can induce NET formation in neutrophils (31, 32). These findings suggest that flavins can directly capture electrons excited by visible and invisible light spectrum to generate some ROS. Nevertheless, since mitoROS inhibitors drastically suppress NETosis, mitoROS is the major contributor of UV-induced NETosis (**Figures 1**, **S1-4**). UV generates free electrons that can be captured by flavins in neutrophils (31). Mature neutrophil mitochondria primarily contain ROS producing complex II with its flavin adenosine dinucleotide (FAD) co-factor system (33). Complex II along with the FAD system generates ROS in neutrophils (34). Inhibition of complex II disrupted ROS production by UV radiation (17). Therefore, UV likely uses this system to produce mitochondrial ROS in neutrophils. This confirms that UV induces MitoROS-dependent NOX-independent NETosis (7). UV is known to modify the enzymes to increase ROS (30). Our study shows that UV irradiation leads to increased ROS production and DNA oxidation (e.g., mitoROS, 8-oxoG; **Figures 1**, **2**). When the Redox balance is altered by increasing ROS scavengers, the DNA-damaging activity of ROS may be reduced. Serum components often reduce ROS levels (14, 35), and hence, the effect of UV on NETosis may be different under different tissue conditions. In general, mitoROS generation and NOX-independent NETosis have been reported during other sterile injuries (e.g., autoimmunity in Lupus, urate crystals in gout) (36–38). Hence, the pathway discovered in this study may be applicable to some of the other sterile injury conditions as well.

Neutrophils possess intricate mitochondrial networks capable of generating substantial ROS (39). The absence of significant levels of cellular ROS, despite the presence of mitoROS and DNA damage, may be attributed to the mitochondrial retrograde response signaling pathway. In times of cellular distress, mitochondria engage in retro-communication with the nucleus, facilitated by direct contact sites established between them. It has been documented that perinuclear clustering of mitochondria takes place in neutrophils (40). This phenomenon could elucidate a potential mechanism by which mitochondrial ROS accumulation leads to nuclear DNA damage.

As mentioned above, neutrophils carry many pre-synthesized DNA repair proteins, such as OGG1, PCNA, PARP, and DNA Polβ (24–27). The key steps of BER are base removal, incision, end processing, repair synthesis and ligation (15, 28). Our data indicate that inhibiting the assembly of DNA repair enzyme complexes and nicking of DNA strands at the early steps of BER (APE1, PARP1, DNA ligase) suppresses UV-induced NETosis (**Figure 3**). Hence, DNA repair activation is necessary for NETosis to occur. Despite carrying out its role in the last stage of BER, DNA ligase is part of the early complex and is necessary for the assembly of the repair initiating machinery (29). One possible explanation is that by requiring DNA ligase to be part of the initiating complex, BER ensures that the nicks are effectively sealed after the repair. Our results indicate that PCNA is found bound throughout the NET DNA (**Figure 3**). This suggests that DNA damage and repair are distributed across all regions of the genome. Previous studies suggested that the purpose of the stored PCNA is to regulate apoptosis (24). Here, we suggest that PCNA is participating in DNA repair. Our recent study shows that ROS and early stage BER proteins are important for inducing spontaneous NETosis; late stage BER proteins (PCNA, Polβ, Polδ) repair the baseline oxidative damage and suppress such NETosis, whereas inhibition of these latter repair steps promotes NETosis (13, 14). Nevertheless, inhibiting the later stages of BER did not affect UV-induced NETosis. We have seen a similar type of inhibition by BER inhibitors when high level of ROS was generated in NOX-dependent NETosis induced by PMA, LPS or bacteria (13). This is likely because these proteins are not involved in the unwinding and nicking of the chromatin (29). Once DNA is excessively oxidized by large amounts of ROS, generated either by NOX or mitochondria, and chromatin is decondensed by nicking/unwinding, the effect of PCNA and repair synthesis steps will have limited ability to suppress NETosis. Hence, the role of late-stage proteins in NETosis is dependent on the context. UV can also generate cyclobutane pyrimidine dimers and 6-4 photoproducts that are repaired by nucleotide excision repair pathway (41). Since BER pathway enzyme inhibitors (e.g., APE1) drastically suppress UV-induced NETosis, BER plays a major role in NETosis.

We have previously shown that kinase activation and transcription also contribute to NETosis, including UV-induced NETosis (7, 42). Combining our previous discoveries with our most recent one, a logical model is that kinases activate transcription factors, which results in the activation of transcription. Transcription machinery stalls at sites of oxidative DNA damage and effectively recruits DNA repair machinery, a phenomenon previously uncovered by others (43, 44). The repair machinery acts at the sites to unwind the chromatin and nick the DNA, creating a temporary site of unwound chromatin. As these sites of unwound/nicked DNA accumulate globally, their effects are compounded resulting in the complete opening of the chromatin and NETosis (**Figure 4**).

The ozone layer plays a critical role in protecting life on Earth from harmful ultraviolet radiation. UV-C radiation is the most energetic and harmful form of UV radiation, and is largely absorbed by the ozone layer in the Earth’s atmosphere. However, when the ozone layer is depleted over the arctic, at high altitudes and during severe global warming events, more UV radiation can penetrate the atmosphere and reach the Earth’s surface (45), such as during the presence of the ozone hole (46). This increased UV-C exposure can have damaging effects on living organisms, including increased risk of skin cancer, damage to DNA, and harm to the immune system. Humans can also be exposed to UV-C radiation through certain artificial sources, such as mercury vapor lamps, some types of welding equipment, and certain types of sterilization lamps used in medical and laboratory settings, such as during medical accidents (47). Furthermore, UV exposure has been increasing and auto-immune diseases, such as lupus, are becoming increasingly prevalent (48). UV can also act as a key inducer of damage to skin (47, 49, 50). UV exposure is known to contribute/exacerbate these and many other diseases; hence, a better understanding of UV-induced NETosis will help to uncover novel mutations that could alter the susceptibility to autoimmune diseases, and identify targets to devise therapeutic treatments.

## Data availability statement

The raw data supporting the conclusions of this article will be made available by the authors, without undue reservation.

## Ethics statement

The studies involving human participants were reviewed and approved by The Hospital for Sick Children Research Ethics Committee. The patients/participants provided their written informed consent to participate in this study.

## Author contributions

D.A conceived the study, planned/conducted the experiments, analyzed the data, generated figures and drafted the manuscript. N.P is the principal investigator, conceived the study, provided feedback, and edited and finalized the manuscript.
