# Supplementary material for: Mitochondrial ROS and base excision repair steps leading to DNA nick formation drive ultraviolet induced-NETosis
Source: Front Immunol. 2023 Jun 7;14:1198716. doi: 10.3389/fimmu.2023.1198716 (PMC10282603; doi:10.3389/fimmu.2023.1198716)
Supplement: Supplementary file 1 [file DataSheet_1.pdf]

Supplemental Figures:

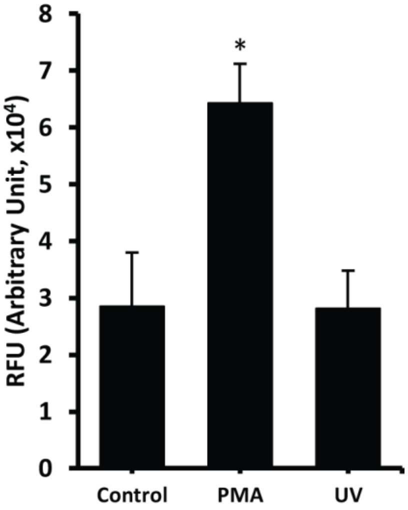

Figure S1: PMA, but not UV, induces NOX ROS. Neutrophils were treated with media, PMA or UV, and then incubated with ROS detection fluorescence probe DHR123. The ROS-oxidized probe was quantified using plate reader assays (R123 fluorescence; n = 3; *, p<0.05 compared to media control, One-way ANOVA with Dunnett's multiple comparison test).

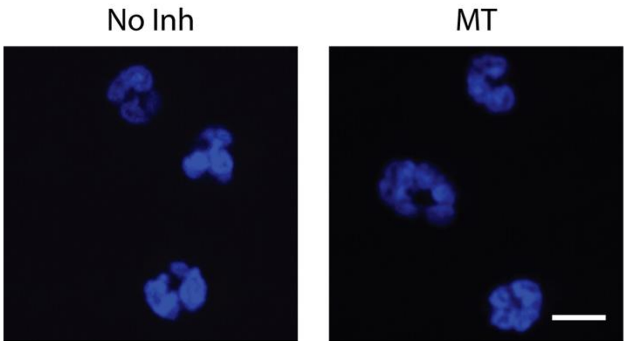

Figure S2. MitoTempo does not induce apoptosis in neutrophils within 4 hours. Neutrophils were incubated with media or MitoTempo (100 µM) and incubated for 240 min. Cells were stained for DNA (DAPI, blue). Images are representative of three independent experiments (scale bar, 10 µm).

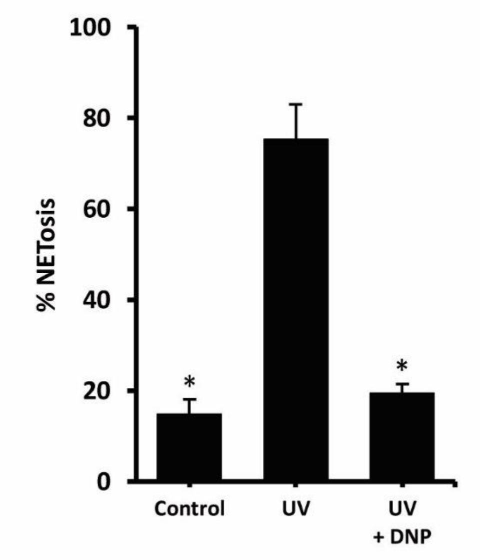

Figure S3. UV induces mitochondrial ROS-mediated NETosis. DNA release following UV (1.92 J/cm^2) exposure was measured using SYTOX Green plate reader assay (n = 3; error bars represent SEM; *$p < 0.05$ compared to the cells treated with UV alone; One-way ANOVA with Dunnett's multiple comparison test). Cells were preincubated with DNP (750 μM) for 1-h prior to UV treatment. DNP significantly inhibits UV-induced NETosis.

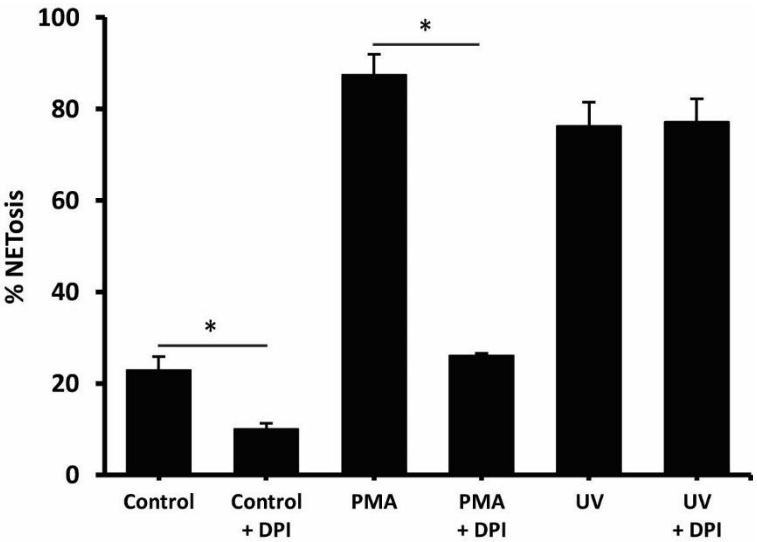

Figure S4. UV induces NOX-independent NETosis. DNA release following UV (1.92 J/cm^2) exposure was measured using SYTOX Green plate reader assay (n = 3; error bars represent SEM; *$p < 0.05$; Two-way ANOVA with Tukey's post test). Cells were preincubated with DPI (1 μM) for 1-h prior to UV treatment. DPI significantly inhibits PMA-induced NETosis but not UV-induced NETosis.
